# Dual-task turn velocity – a novel digital biomarker for mild cognitive impairment and dementia

**DOI:** 10.3389/fnagi.2024.1304265

**Published:** 2024-02-27

**Authors:** Jing Wang, Zheping Zhou, Shanshan Cheng, Li Zhou, Xiaoou Sun, Ziyang Song, Zhiwei Wu, Jinhua Lu, Yiren Qin, Yueju Wang

**Affiliations:** ^1^Department of Geriatrics, The First Affiliated Hospital of Soochow University, Suzhou, China; ^2^Department of Geriatrics, Affiliated Changshu Hospital of Nantong University, Changshu, China; ^3^Department of Nutritional Medicine, The First Affiliated Hospital of Soochow University, Suzhou, China; ^4^Department of Neurosurgery, The First Affiliated Hospital of Soochow University, Suzhou, China; ^5^Department of Radiology, The First Affiliated Hospital of Soochow University, Suzhou, China; ^6^Department of Neurology, The First Affiliated Hospital of Soochow University, Suzhou, China

**Keywords:** cognitive impairment, turn velocity, dual-task gait, digital biomarker, wearable device

## Abstract

**Background:**

Disorders associated with cognitive impairment impose a significant burden on both families and society. Previous studies have indicated that gait characteristics under dual-task as reliable markers of early cognitive impairment. Therefore, digital gait detection has great potential for future cognitive screening. However, research on digital biomarkers based on smart devices to identify cognitive impairment remains limited. The aim of this study is to explore digital gait biomarkers by utilizing intelligent wearable devices for discriminating mild cognitive impairment and dementia.

**Methods:**

This study included 122 subjects (age: 74.7 ± 7.7 years) diagnosed with normal cognition (NC, *n* = 38), mild cognitive impairment (MCI, *n* = 42), or dementia (*n* = 42). All subjects underwent comprehensive neuropsychological assessments and cranial Magnetic Resonance Imaging (MRI). Gait parameters were collected using validated wearable devices in both single-task and dual-task (DT). We analyzed the ability of gait variables to predict MCI and dementia, and examined the correlations between specific DT-gait parameters and sub-cognitive functions as well as hippocampal atrophy.

**Results:**

Our results demonstrated that dual-task could significantly improve the ability to predict cognitive impairment based on gait parameters such as gait speed (GS) and stride length (SL). Additionally, we discovered that turn velocity (TV and DT-TV) can be a valuable novel digital marker for predicting MCI and dementia, for identifying MCI (DT-TV: AUC = 0.801, sensitivity 0.738, specificity 0.842), and dementia (DT-TV: AUC = 0.923, sensitivity 0.857, specificity 0.842). The correlation analysis and linear regression analysis revealed a robust association between DT-TV and memory function, as well as the hippocampus atrophy.

**Conclusion:**

This study presents a novel finding that DT-TV could accurately identify varying degrees of cognitive impairment. DT-TV is strongly correlated with memory function and hippocampus shrinkage, suggests that it can accurately reflect changes in cognitive function. Therefore, DT-TV could serve as a novel and effective digital biomarker for discriminating cognitive impairment.

## 1 Introduction

With the coming acceleration of global population aging, it is expected that the number of dementia patients worldwide will triple by 2050 [American Psychiatric Association (APA), 2013; Organization, W.H, 2021], which will place a heavy burden on families and nations. Mild cognitive impairment (MCI) serves as an intermediary state between healthy aging and the initial phases of dementia (Petersen, 2004). Consequently, timely identification and intervention to impede the progression of MCI towards dementia assume paramount importance. Currently, the assessment and detection of dementia are time-consuming and laborious, highlighting the urgent need for convenient screening methods. Previous studies have pointed out that gait disturbances are highly probable to function as an initial screening indicator for MCI (Waite et al., 2005; Garcia-Pinillos et al., 2016; Ramirez and Gutierrez, 2021). Therefore, the search for digital gait markers holds significant importance for future cognitive impairment screening.

Early gait studies on MCI or AD (Alzheimer’s disease) have primarily utilized a single-task paradigm, recent researchers have proposed a dual-task paradigm that closely resembles daily life. This paradigm involves combining motor tasks (such as walking) with cognitive tasks (particularly those related to executive function, such as naming animals or counting backward). Substantial evidence indicates that individuals with MCI or AD exhibit poorer gait performance under dual-task conditions, including slower gait, increased gait instability and variability (Oh, 2021; Ramirez and Gutierrez, 2021). Systematic reviews and meta-analyses conducted by Bahureksa et al. (2017) and Fuentes-Abolafio et al. (2021) have demonstrated that gait speed, stride length, and stride time can accurately differentiate MCI patients from healthy controls, particularly in dual-task conditions. Furthermore, Toulotte et al. (2006) found that dual tasks negatively impacted walking ability in older individuals who had experienced falls, leading to compromised static balance. Shumway-Cook et al. also discovered that balance training under dual-task condition was more effective than single-task training in improving balance and cognitive performance in older adults with balance impairments (Silsupadol et al., 2009). Current neural theories suggest that there may be shared neural networks between motor and cognitive functions, and performing dual tasks may overload these networks (Tombu and Jolicoeur, 2003). AD patients, in particular, have impaired ability to share cognitive resources (Perry and Hodges, 1999; Sheridan et al., 2003). As a result, dual-task gait assessment based on smart and wearable digital technologies holds significant potential for detecting MCI and dementia.

However, the majority of current gait studies on cognitive impairment primarily rely on non-portable gait analysis techniques, which is inconvenient and fails to accurately capture the patient’s gait changes during daily activities, making it unsuitable for home settings. In recent years, portable sensor devices have emerged as a new type of equipment and have been extensively used in studying neurological disorders of gait abnormalities, such as multiple sclerosis, hydrocephalus, as well as Parkinson’s disease. Nevertheless, it is rarely used to analyze gait in individuals with cognitive impairment diseases, such as AD. In limited studies, most have solely focused on fall prognosis (Mancioppi et al., 2021; Weizman et al., 2021).

In studies involving portable devices, these equipment are predominantly designed to detect gait in straight-line walking mode and are limited to tracking the motor performance of the lower limbs (Auvinet et al., 2017), resulting in the gait parameters available are finite. In this study, we are the first to apply the intelligent and portable APDM’s Mobility Lab system for gait detection in patients with cognitive impairment. The device is equipped with 6 sensors that can be utilized in open flat areas. It is capable of collecting data on gait and balance function in both straight and turn modes, which includes complex spatiotemporal gait and kinematic parameters. It automatically analyzes and calculates the collected data, providing gait information upon completion of the detection. In comparison, other portable devices in cognitive study often have only one sensor and can collect limited gait data. Some even require fixed laboratory monitoring, and very few devices are capable of detecting turning data (Fuentes-Abolafio et al., 2020, 2021).

Our primary objective is to utilize intelligent wearables to analyze the changes in gait parameters among patients with MCI and dementia under single-task and dual-task conditions and explore new gait indicators that can distinguish patients with cognitive impairment (MCI and dementia) from normal older adults. Additionally, we will investigate the correlations between these gait prediction indicators and various cognitive domains as well as hippocampal atrophy. We anticipate that the findings from this study will serve as a theoretical foundation for the future application of digital gait parameters detected by smart wearable devices in the screening of MCI and dementia patients.

## 2 Methods

### 2.1 Study design and participants

The old adults with or without cognitive impairment were invited into this study. Old patients with cognitive decline were recruited from the Memory Disorders Clinic of the Department of Geriatrics in the First Affiliated Hospital of Soochow University, and the individuals who were cognitively intact and healthy were invited as control group. All participants had to meet the following criteria: age 60 years and older, able to walk independently for 10 min without gait assistance, and without severe physical disease or contraindications for MRI. The study protocol was approved by the Ethics Committee of the First Affiliated Hospital of Soochow University. Prior to participating in this study, all participants and their caregivers signed informed consent forms.

Exclusion criteria included:(1) having neurological and skeletal muscle diseases that may cause gait abnormalities (such as stroke, Parkinson’s disease, severe arthritis, severe trauma, or surgery to the lower limbs); (2) having severe mental disease (such as people with severe depression, schizophrenia, alcohol use disorder, bipolar disorder, and anxiety disorder, etc., who are unable to cooperate and complete gait and cognitive assessments); (3) severe visual impairment and hearing impairment; (4) subjects with obvious white matter lesions (Fazekas score of 3 or higher).

Then, all participants underwent comprehensive neuropsychological testing and assessment of daily living abilities and were diagnosed with normal cognitive (NC), MCI or dementia by expert neurologists. According to Petersen et al. (2001), if a participant: (a) complaint for cognitive decline; (b) smaller than −1.5 standard deviation from mean of local population in at least one cognitive domain with the standard neuropsychological assessments; (c) preserved basic activities of daily living (ADL) with slightly impaired instrumental activities of daily living (IADL) (Lawton and Brody, 1969); (d) failure to meet the Diagnostic and Statistical Manual of Mental Disorders, 4th edition (DSM-IV); (e) 18 ≤ the Montreal Cognitive Assessment (MoCA) < 26 adjusted and education (Nasreddine et al., 2005), will be diagnosed with MCI. According to the DSM-IV [American Psychiatric Association (APA), 1994], participants who meet the following conditions are diagnosed with dementia. They have previously had normal intelligence and then have acquired cognitive decline (impairment of memory, execution, language, or visuospatial ability) or abnormal mental behavior that interferes with work or daily living and cannot be explained by delirium or other psychiatric disorders, assessed in combination with an objective scale, and has at least 2 of the following 5 items: (a) impaired memory and learning ability; (2) impaired executive functions such as reasoning, judgment and handling of complex tasks; (3) impaired visuospatial ability; (4) impaired language function (listening, speaking, reading, writing); (5) Changes in personality, behavior, or demeanor. The NC group consisted of individuals with normal cognitive performance, independent daily living (MoCA score ≥ 26 adjusted education, and normal ADL and IADL). Subsequently, all subjects underwent gait assessment and cerebral Magnetic Resonance Imaging (MRI) tests.

### 2.2 Sociodemographic characteristics and cognitive assessment

Sociodemographic information including age, gender, height, weight, years of education, and medical history (Hypertension, Diabetes mellitus, Coronary heart disease) was collected through face-to-face interviews. Global cognition was assessed by the Mini-Mental State Examination (MMSE) (Folstein et al., 1975) and the Montreal Cognitive Assessment of Beijing version (MoCA-BJ) (Yu et al., 2012) (1 point is added for participants with less than 12 years of education). The Clock Drawing Test (CDT) and the Stroop Color-Word Test-Victoria version (SCWT-VST) (Spreen and Strauss, 1998) were employed to evaluate spatial–temporal and executive function. Attention function was estimated by the Digit Span Test (DST) (Wechsler, 1955). Memory function was measured using the Auditory Verbal Learning Test-HuaShan version (AVLT-H) (Guo et al., 2007). Animal naming trials were conducted to assess participants’ semantic fluency. To assess the participants’ activities of daily living and independent living skills, we used the Instrumental Activities of Daily Living Scale (Shouxi Tao, 1992). Additionally, Hamilton Anxiety and Depression Rating Scales were used to measure the participants’ mental state (Hamilton, 1959; Hamilton, 1960).

### 2.3 Magnetic resonance imaging data acquisition

MRI scanning of the cerebrum was performed on a GE Signa Hdxt 3.0 T scanner (Signa HDxt, GE Healthcare, Milwaukee, WI, United States) by one experienced physician. 3D-T1WI were as follows: TR = 6.52 ms, TE = 2.80 ms, flip angle = 12°, thickness/gap = 1.0/0 mm, field of view (FOV) = 260 × 260 mm, voxel = 1 × 1 × 1 mm3, and acquisition time = 4.25 min. The scan parameters of the T2-FLAIR-weighted imaging were as follows: TR = 8,000 ms, TE = 147 ms, TI = 1,500 ms, thickness/gap = 5.0/1.5 mm, FOV = 240 × 240 mm, and matrix = 256 × 192. Two experienced radiologists used two visual rating scales to identify White matter hyperintensity (WMH) and hippocampal atrophy on baseline images in each subject without knowing the clinical information of the subjects (In the event of disagreement, take the mean of the results of the two assessments). Fazekas scale was used for quantification of WMH, the medial temporal lobe atrophy rating scale (MTA) score was used to judge the degree of bilateral hippocampal atrophy (Fazekas et al., 1987; Scheltens et al., 1995). Finally, patients with severe cerebral small vessel disease (based on the detailed rules of the CSVD Global Burden Rating Scale proposed by Klarenbeek et al. (2013) and Staals et al. (2014), a score of 3 or more was considered), severe cerebral infarction (large-scale cerebral infarction, acute cerebral infarction, and old infarction malacing foci, and the infarction range is more than 1.5 cm), subdural effusion, and hydrocephalus were excluded.

### 2.4 Gait assessment

The gait parameters under single- and dual-task conditions were collected by the wireless APDM Movement Monitoring inertial sensor system (APDM Inc., Portland, OR, United States) which assesses spatial and temporal gait characteristics. APDM’s Mobility Lab^™^ (APDM, Inc) (Mancini et al., 2011) automatically analyzed gait feature. Six Opals were worn on the body of subjects (one on the low back, two on the shanks, one on the sternum, and two on the wrists, see Supplementary material S1). Subjects were instructed to walk at their usual velocity with comfortable shoes on a 7-meter-long straight sidewalk (marked with colored tape at the start and end points), turn 180°, and return to the starting point. This entire process lasted for 2 min. Before conducting the trials, the subjects were given standard guidance and demonstrations. In the single-task test, subjects were asked to walk at their usual pace. In the dual-task condition, they were instructed to name as many different animals as possible while walking at their usual speed (All participants were not informed in advance of the need to name the animal). An experienced geriatrician walked behind the subjects to prevent falls. We selected 9 gait parameters of interest for further analysis in each condition. Supplementary material S2 provides an explanation of all gait parameters. In the end, a total of 122 subjects successfully completed all the evaluations.

### 2.5 Statistical analysis

All data and analyses were performed using SPSS for Windows Version 23.0 (IBM Corp., NY, United States). Shapiro–Wilk test assessed the normality of distributions. The data obtained from our study met the assumption of normal distribution for tests. Mean ± standard deviation was used to present continuous data, while percentages were used for categorical data. Differences in sociodemographic features, clinical features, and gait characteristics among dementia, MCI, and NC subjects were examined using one-way ANOVA with the Bonferroni test as a post-hoc analysis (for parametric variables) or the Kruskal–Wallis test (for non-parametric variables). Categorical variables were analyzed using the χ2 test. To examine the relationship between specific gait parameters and different cognitive functions, we standardized the comprehensive scores of neuropsychological tests for each cognitive domain (The test results of each cognitive domain were summed separately and converted into Z-scores). We then conducted multiple linear regression and partial Spearman rank order correlation analysis, while adjusting for classical confounders such as age and educational level. Additionally, the Person product–moment correlation was used to analyze the associations between specific DT gait indicators with the MTA score of the hippocampus. Univariate logistic regression analysis was performed for all gait parameters. To determine the accuracy of gait parameters in distinguishing between dementia, MCI, and NC groups, we analyzed the area under the curve (AUC) of the Receiver Operating Characteristics (ROC).

## 3 Results

### 3.1 Participant characteristics

In this study, a total of 122 older adults (mean age 74.7 ± 7.7 years) were included. The participants were diagnosed with dementia (*n* = 42), MCI (*n* = 42), or normal cognitive function (NC) (*n* = 38) respectively. The sociodemographic and medical characteristics of participants are presented in Table 1. Age, years of education showed significant differences between the three groups (*p* < 0.05). No significant differences were found in the proportion of sex, height, BMI, hypertension, diabetes, coronary heart disease, anxiety, and depression scores between the three groups. Compared to the NC individuals, the dementia participants exhibited greater difficulty with instrumental ADL. The differences of global cognitive function and sub-cognitive functions (AVLT-H, DT span, CDT, SCWT, and the average number of Animal Naming) across the three groups were statistically significant.

**Table 1:** Demographics and clinical characteristics (*N* = 122).

Variable	NC (*n* = 38)	MCI (*n* = 42)	Dementia (*n* = 42)
Demographics and clinical characteristics			
Age, years	70.6 (7.0)	75.7 (7.2)*	76.9 (7.7)*
Male, *n* (%)	24 (63.2%)	23 (54.8%)	17 (40.5%)
Female (%)	14 (36.8%)	19 (45.2%)	25 (59.5%)
Education, years	10.9 (3.5)	8.8 (4.1)*	5.1 (5.0)*†
BMI (kg/m^2^)	23.1 (3.3)	22.9 (4.4)	23.1 (7.2)
Height (m)	1.64 (0.08)	1.61 (0.08)	1.60 (0.08)
Hypertension, *n* (%)	13 (34.2%)	19 (45.2%)	13 (28.6%)
Diabetes mellitus, (*n*%)	7 (18.4%)	6 (14.3%)	10 (23.8%)
Coronary heart disease, (*n*%)	1 (2.6%)	2 (4.8%)	3 (7.1%)
MMSE score	27.6 (1.6)	23.1 (3.2)*	12.1 (4.2)*†
MoCA score	26.2 (2.6)	19.7 (2.9)*	9.9 (3.2)*†
Hamilton anxiety scale	3.9 (3.8)	2.5 (2.3) *	2.9 (3.1)
Hamilton depression scale	3.5 (4.3)	2.8 (2.9)	2.9 (3.9)
ADL	8.0 (0.0)	9.1 (2.8)	10.0 (4.5)*
IADL	12.2 (0.6)	15.7 (7.2)*	17.7 (8.5)*
AVLT-H (Immediate recall)^#^	16.2 (4.1)	11.3 (4.4)*	4.9 (4.5)*†
DST (Forward) ^#^	7.9 (0.8)	7.1 (1.0)*	5.8 (1.2)*†
Animal Naming	14.7 (2.8)	12 (3.6)*	6.9 (3.6)*†
CDT	3.9 (0.2)	2.7 (1.1)*	1.2 (0.8)*†
SCWT (Color (s)) ^#^	39.7 (1.9)	56.2 (30.8)*	62.9 (24.7)*

NC: normal cognitive; MCI: mild cognitive impairment; BMI: body mass index; MMSE: mini-mental state examination; MoCA: montreal cognitive assessment; ADL/IADL: instrumental/activity of daily living; AVLT-H: auditory verbal learning test-HuaShan version; DST: digital span test; CDT: clock drawing test; SCWT: stroop color word test;*p<0.05, versus NC; †*p*<0.05, versus MCI; ^#^ See Supplementary material S7 for more details.

### 3.2 Gait characteristics between the groups under the single- and dual-task walking

According to Table 2, in both single- and dual-task conditions, individuals with cognitive impairment performed worse in overall gait compared to NC participants. In single-task test, subjects with dementia and MCI presented with slower gait speed (GS: 63.5 ± 18.9 cm/s vs. 71.8 ± 16.6 cm/s vs. 83.2 ± 13.3 cm/s), slower turn velocity (TV: 123.9 ± 29.9 degree/s vs. 138.0 ± 31.4 degree/s vs. 165.3 ± 30.0 degree/s), shorter stride length (SL: 75.7 ± 19.3 cm vs. 83.3 ± 17.3 cm vs. 97.8 ± 13.5 cm) than NC group, and the differences were found to be statistically significant among the three groups. Furthermore, as the degree of cognitive impairment increased, individuals tended to have longer double support and standing phases, shorter swing phases, and smaller lateral step variability. However, significant differences were only observed between the NC group and the dementia group (*p <* 0.05). Under dual-task gait assessment, the dementia and MCI patients exhibited significantly slower GS and TV, as well as significantly shorter SL than NC group (DT-GS: 42.6 ± 14.4 cm/s vs. 48.8 ± 14.3 cm/s vs. 65.2 ± 11.3 cm/s; DT-TV: 101.3 ± 23.0 degree/s vs. 117.9 ± 29.1 degree/s vs. 151.0 ± 38.5 degree/s; DT-SL: 58.5 ± 16.7 cm vs. 66.4 ± 15.9 cm vs. 86.9 ± 11.8 cm, see Supplementary material S3), the post-hoc test also revealed significant differences between the three groups. Additionally, there were significant differences observed in double support, standing phases, swing phases, and lateral step variability not only between the dementia and NC groups, but also between the MCI and NC groups (*p*<0.05). Either in single or dual-task, there was no significant difference in cadence between the three groups. Whatever the condition, the GS, SL, and TV always presented significant differences among the three groups.

**Table 2:** Gait parameters in single- and dual-task (*N* = 122).

Variable	NC (*n* = 38)	MCI (*n* = 42)	Dementia (*n* = 42)
Single-task ^#^			
Gait speed (cm/s)	83.2 (13.3)	71.8 (16.6)*	63.5 (18.9)*†
Stride length (cm)	97.8 (13.5)	83.3 (17.3)*	75.7 (19.3)*†
Turn velocity (degrees/s)	165.3 (30.0)	138.0 (31.4)*	123.9 (29.9)*†
Dual-task			
Gait speed (cm/s)	65.2 (11.3)	48.8 (14.3)*	42.6 (14.4)*†
Cadence (steps/min)	90.3 (12.8)	88.8 (16.5)	89.1 (15.7)
Stride length (cm)	86.9 (11.8)	66.4 (15.9)*	58.5 (16.7)*†
Double support L (%GCT)	26.7 (3.4)	30.7 (6.0)*	32.2 (6.8)*
Double support R (%GCT)	26.6 (3.3)	31.0 (5.8)*	33.1 (8.1)*
Lateral step variability (cm)	7.7 (2.7)	5.6 (2.7)*	5.1 (2.4)*
Stance (%GCT)	63.3 (1.7)	65.8 (2.9)*	66.5 (4.2)*
Swing (%GCT)	36.7 (1.7)	34.5 (2.9)*	33.7 (3.5)*
Turn velocity (degrees/s)	151.0 (38.5)	117.9 (29.1)*	101.3 (23.0)*†

NC: normal cognitive; MCI: mild cognitive impairment;*p<0.05, versus NC; †*p*<0.05, versus MCI; ^#^ See Supplementary material S7 for more details.

### 3.3 Univariate logistic regression

As shown in Table 3, univariate logistic regression analysis was used for data processing, after adjusting for confounders (age, years of education). The results showed that, compared to single-task, almost all DT parameters were associated with an increased risk of cognitive impairment, reaching statistical significance (such as the longer double support times, shorter swing phases, and smaller lateral step variability). The gait features that were more significantly different between the three groups under two different task conditions were GS, TV, and SL, these three parameters were found to increase the risk of MCI and dementia, with statistical significance, each 1 cm/s increase in DT-GS decreased the risk of MCI by 18.3% and the risk of dementia by 26.2%, each 1 degree/s increase in DT-TV decreased the risk of MCI by 4.5%and the risk of dementia by 9.3%, each 1 cm increase in DT-SL decreased the risk of MCI by 14.9% and the risk of dementia by 24.8%.

**Table 3:** Logistics regression analysis of gait characteristics (NC-MCI & NC-Dementia).

	NC-MCI			NC-Dementia		
Variable	OR	*P*	95%CI	OR	*P*	95%CI
Single task						
Gait speed	0.943	0.006	0.904–0.983	0.934	0.008	0.888–0.982
Stride length	0.938	0.003	0.898–0.979	0.925	0.006	0.875–0.978
Double support L	1.193	0.024	1.023–1.390	1.149	0.077	0.985–1.340
Double support R	1.187	0.032	1.015–1.390	1.152	0.072	0.988–1.343
Lateral step variability	0.853	0.073	0.717–1.015	0.808	0.058	0.646–1.001
Stance	1.390	0.036	1.022–1.890	1.350	0.055	0.994–1.832
Swing	0.720	0.036	0.529–0.978	0.741	0.055	0.546–1.006
Turn velocity	0.975	0.007	0.958–0.993	0.967	0.005	0.945–0.990
Dual-task						
Gait speed	0.817	0.000	0.734–0.910	0.738	0.001	0.615–0.885
Stride length	0.851	0.000	0.782–0.926	0.752	0.001	0.632–0.894
Double support L	1.359	0.001	1.130–1.634	1.305	0.004	1.088–1.565
Double support R	1.431	0.000	1.171–1.749	1.368	0.002	1.123–1.666
Lateral step variability	0.762	0.008	0.624–0.931	0.692	0.006	0.534–0.898
Stance	1.945	0.001	1.328–2.8647	1.741	0.003	1.211–2.504
Swing	0.515	0.001	0.352–0.753	0.574	0.003	0.400–0.825
Turn velocity	0.955	0.000	0.932–0.979	0.907	0.000	0.866–0.950

Adjusted for age, sex, years of education.

### 3.4 Receiver operating characteristics curves

**Figure 1:**
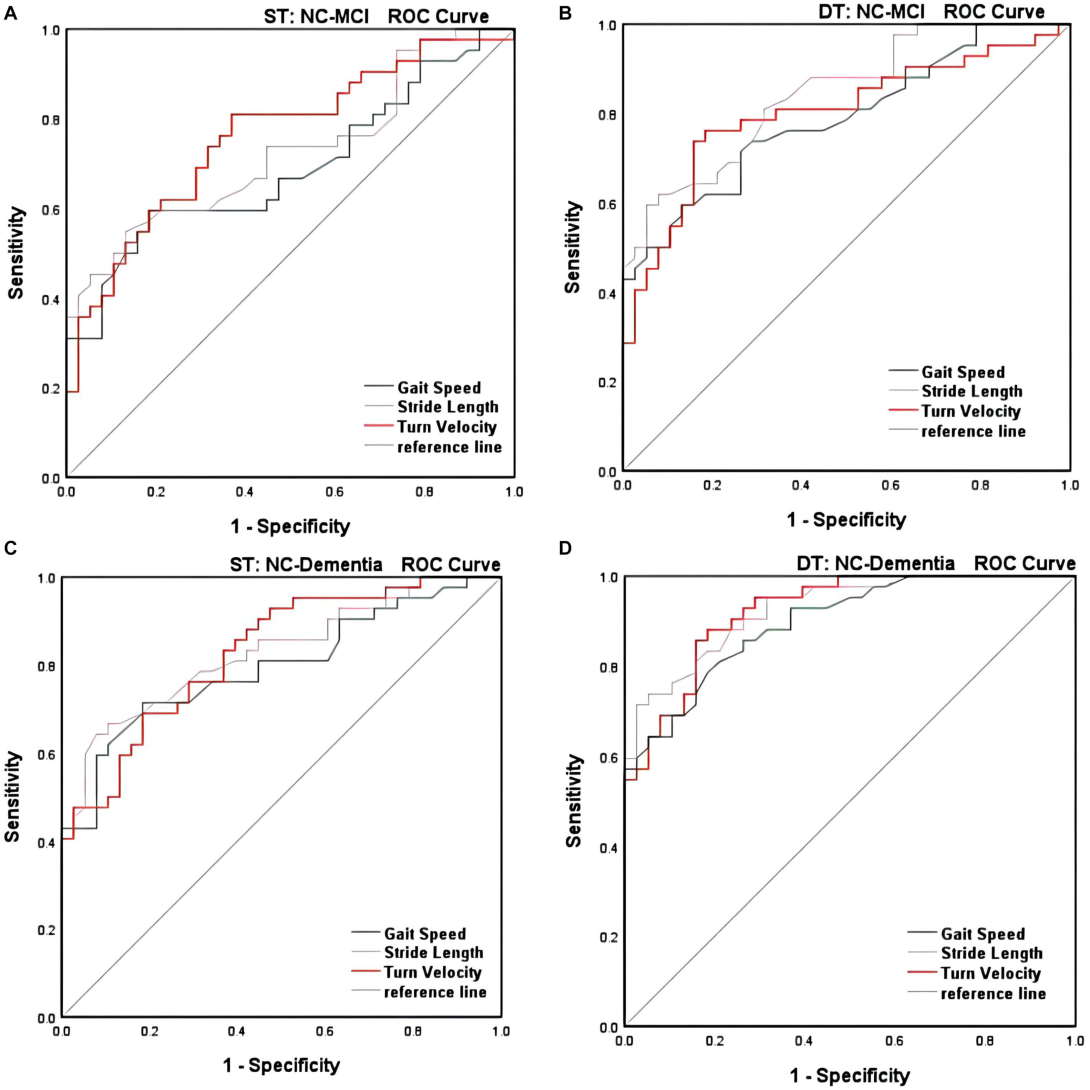
**(A)** Global performance of gait parameters under single-task walking for identification of NC with MCI. **(B)** Global performance of gait parameters under dual-task walking for identification of NC with MCI. **(C)** Global perfonnance of gait parameters under single-task walking for identification of NC with dementia. **(D)** Global perfonnance of gait parameters under dual-task walking for identification of NC with dementia.

As shown in Table 4, we examined the predictive power of different gait parameters for varying degrees of cognitive impairment during different tasks (in previous univariate logistic analyses, they were found to independently affect MCI or dementia). For single-task walking, three characteristics including GS (AUC = 0.689, sensitivity = 59.5%, specificity = 81.5%), SL (AUC = 0.726, sensitivity = 54.8%, specificity = 86.8%), and TV (AUC = 0.761, sensitivity = 81.0%, specificity = 63.1%) were able to discriminate reasonably well between the NC and MCI groups. However, under dual-task conditions, the discrimination ability of all gait characteristics significantly improved, such as DT-GS (AUC = 0.788, sensitivity = 59.5%, specificity = 86.8%), DT-SL (AUC = 0.844, sensitivity = 59.5%, specificity = 94.7%), DT-TV (AUC = 0.801, sensitivity = 73.8%, specificity = 84.2%), DT-lateral step variability (AUC = 0.721, sensitivity = 81.0%, specificity = 65.8%). Moreover, when differentiating between the NC and dementia groups, no matter in the case of single- or dual-task, the discrimination ability of GS, SL and TV are excellent, especially DT-GS (AUC = 0.897, sensitivity = 78.6%, and specificity = 81.5%), DT-SL (AUC = 0.926, sensitivity = 71.4%, and specificity = 97.4%), DT-TV (AUC = 0.923, sensitivity = 85.7%, and specificity = 84.2%). Our findings consistently demonstrate that DT-GS, DT-SL, and DT-TV outperformed other gait indexes in terms of prediction ability. Additionally, the predictive model for TV exhibited high sensitivity and specificity in both single- and dual-task conditions (see Figure 1).

**Table 4:** Area under the curve (AUC) and 95% confidence interval (CI) of gait variables between the groups during single- and dual-task walking (NC vs. MCI & NC vs. Dementia).

Variable	AUC	95%CI	Sensitivity	Specificity	AUC	95%CI	Sensitivity	Specificity
	NC vs. MCI				NC vs. Dementia			
Single task								
Gait speed	0.689	0.572–0.806	0.595	0.815	0.796	0.698–0.894	0.714	0.815
Stride length	0.726	0.615–0.836	0.548	0.868	0.829	0.740–0.918	0.643	0.921
Turn velocity	0.761	0.657–0.866	0.810	0.631	0.829	0.742–0.916	0.690	0.815
Dual task								
Gait speed	0.788	0.690–0.886	0.595	0.868	0.897	0.832–0.962	0.786	0.815
Stride length	0.844	0.761–0.926	0.595	0.947	0.926	0.873–0.979	0.714	0.974
Double support L	0.704	0.588–0.820	0.548	0.868	0.747	0.640–0.854	0.571	0.895
Double support R	0.735	0.625–0.845	0.571	0.868	0.770	0.667–0.874	0.524	0.947
Lateral step variability	0.721	0.608–0.835	0.810	0.658	0.755	0.649–0.861	0.786	0.657
Stance	0.723	0.611–0.835	0.476	0.947	0.759	0.654–0.864	0.571	0.947
Swing	0.727	0.615–0.838	0.476	0.947	0.763	0.659–0.868	0.571	0.947
Turn velocity	0.801	0.703–0.900	0.738	0.842	0.923	0.868–0.977	0.857	0.842

### 3.5 Correlations of different cognitive functions with dual-task gait characteristics

**Figure 2:**
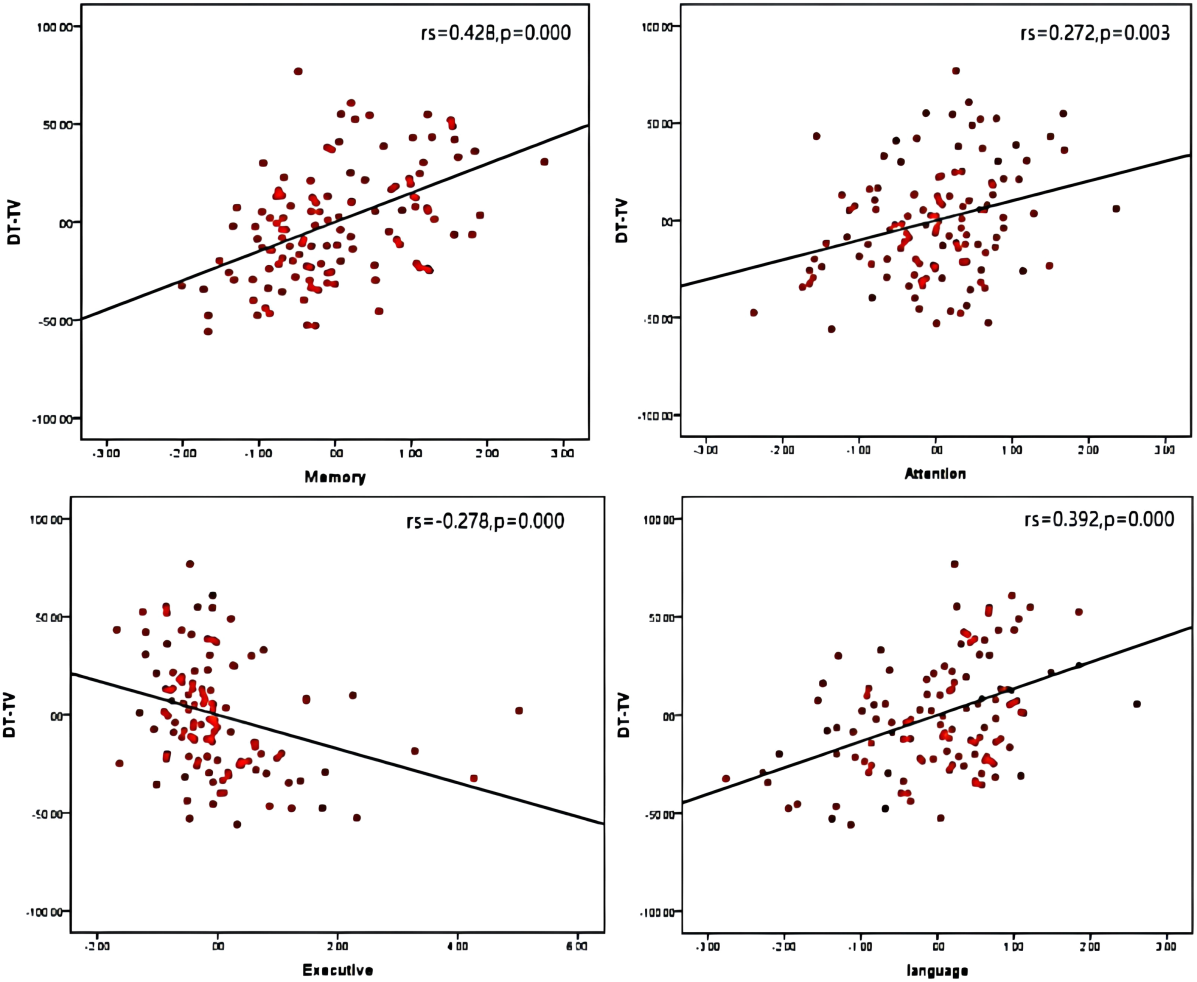
Correlations between the turn velocity under dual-task walking (DT-TV) with different cognitive function.

We examined correlations of DT-GS, DT-SL, and DT-TV with global cognitive function and sub-cognitive functions separately. Age, sex, and years of education were corrected during examination. Then, strong correlations were observed between the three indicators and MoCA (see Supplementary material S4), DT-TV exhibited the strongest correlation with memory, executive function, and language function (memory: r_s_ = 0.428, *p* < 0.01; executive: r_s_ = −0.278, *p* < 0.01; language: r_s_ = 0.392, *p* < 0.01; see Figure 2) compared to the other two indicators, DT-SL showed the strongest correlation with attention function (attention: r_s_ = 0.347, *p* < 0.01). However, the correlation between DT-GS and each cognitive domain was not prominent.

As we observed significant correlations between sub-cognitive function with DT-gait parameters. To assess the potential impact of sub-cognitive function on DT gait performance, we conducted a linear regression analysis (refer to Supplementary material S5). Our findings indicate that only memory function had a significant effect on DT-GS (β = 0.322, *p* = 0.003), DT-SL (β = 0.274, *p* = 0.007) and DT-TV (β = 0.285, *p* = 0.006) individually.

### 3.6 Correlations of MTA grade of the hippocampus with dual-task gait characteristics

Subsequently, we conducted an analysis on the correlations between DT-gait characteristics and the MTA grade of the hippocampus (see Supplementary material S6). The findings revealed a negative correlation between the DT-gait parameters and hippocampus atrophy. Notably, the correlation was more pronounced on the right side compared to the left. Moreover, we observed that the correlations between hippocampus atrophy and stride length, as well as turn velocity, was stronger than that with gait speed (DT-GS r_s_ = −0.264, *p* < 0.01; DT-SL r_s_ = −0.406, *p* < 0.01; DT-TV r_s_ = −0.381, *p* < 0.01).

## 4 Discussion

Nowadays, the development of convenient and efficient digital wearable device technology is continuously progressing, playing an increasingly important role in the early screening of neurodegenerative diseases (Kourtis et al., 2019). In this study, we have applied intelligent devices (APDM Movement Monitoring inertial sensor system) for the first time to gait detection in patients with MCI and dementia. Our results suggested that gait parameters obtained through the intelligent sensor device can more accurately differentiate patients with MCI and dementia from cognitively healthy individuals. Furthermore, we confirmed that indicators during dual-task walking were preferable, aligning with previous research (MacAulay et al., 2017; Oh, 2021). Among these gait parameters, gait speed and stride length have consistently emerged as prominent indicators, which is in line with numerous previous studies (Montero-Odasso et al., 2014; Beauchet et al., 2018). However, our study has made a novel discovery—turn velocity (TV)—which has shown remarkable ability in accurately distinguishing between normal and cognitive decline groups, and balanced both higher sensitivity and specificity, compared to gait speed and stride length. In addition, we further confirmed that DT-TV was strongly correlated with memory and the degree of hippocampal atrophy. These significant findings highlight the potential for intelligent wearable devices to be utilized in the future for screening individuals with MCI and dementia.

In previous studies, wearable devices are rarely used to assess the gait changes of patients with cognitive impairment, and as a result of technical limitations, these studies were unable to collect indicators related to turning function. The APDM gait analysis system overcomes these limitations by comprehensively acquiring data in both straight walking and turning modes. While it has been widely used in neurological disorders with gait abnormalities such as Parkinson’s and multiple sclerosis, there is limited literature available on its application in gait analysis for diseases related to cognitive impairment. In our study, we leveraged the APDM gait analysis system and found that DT-TV exhibited a particularly excellent performance, which had a high predictive value for MCI and dementia (DT-TV for MCI, AUC 0.801, sensitivity 0.738, specificity 0.842; DT-TV for dementia, AUC 0.923, sensitivity 0.857, specificity 0.842).

Turning is a complex motor pattern which involves more coordination between limbs, coupling between posture and gait, and modifications of movement patterns (Patla et al., 1999), as well as requires higher cognitive demands than straight-walking (Mancini et al., 2016). Previous studies have reported that higher prefrontal cortex activity is strongly linked to poorer turning performance in patients with neurological disorders (Belluscio et al., 2019; Stuart et al., 2019). However, only a few studies that have indirectly analyzed the association between turning function and cognitive impairment. A recent study found that turning function in patients with chronic stroke was associated with cognitive impairment (Kuan et al., 2022). Mirelman et al. (2014) also discovered that individuals with MCI had lower trunk angular velocity during turning. Although no studies have directly investigated turn velocity in patients with MCI and dementia, our results firstly suggest that turn velocity could potentially serve as a novel predictor of MCI and dementia.

Specifically, our study found that DT-GS, DT-SL, and DT-TV parameters showed approximately a 10% improvement in predicting MCI and dementia compared to single-task (in terms of AUC). This improvement is higher than what has been reported in other studies, where typically only about a 6% improvement is observed (Wang X. et al., 2023; Wang Y. et al., 2023). Moreover, our study demonstrated that dual-task gait parameters collected using smart gait devices achieved a dementia prediction rate of up to 90%, highlighting the potential of wearable devices in screening for dementia.

Previous longitudinal studies have demonstrated that executive function and episodic memory have a significant impact on gait speed (Holtzer et al., 2012a; Taylor et al., 2017), and it has been observed that better executive attention is associated with longer stride length (MacAulay et al., 2015). Recent evidence suggests that, in addition to GS and SL, gait parameters related to turning are highly correlated with executive and attention function (Morris et al., 2019; Sunderaraman et al., 2019). In our study, we observed that GS, SL, and TV were all associated with multiple cognitive functions (memory, executive, attention, and language). However, when conducting linear regression analysis, we observed significant correlations only between these three gait measures and memory function. This finding may be attributed to the more detailed assessment of memory function employed in our study. Similar regression analyses conducted in other studies have also reported a strong association between DT-GS and memory function (Holtzer et al., 2012b; Doi et al., 2014). Consequently, individuals with memory impairment may exhibit specific changes in dual-task gait performance.

The association between hippocampal atrophy and decline in gait speed in patients with cognitive impairment has been confirmed by several studies (Callisaya et al., 2013; Rosso et al., 2017; Pieruccini-Faria et al., 2023). Our study further confirmed that strong associations between hippocampal atrophy and decline in DT-GS, DT-SL and DT-TV. The strong correlations between DT-TV and memory function, along with hippocampal atrophy, which are consistent with GS and SL, suggest that DT-TV could serve as a significant indicator of cognitive impairment, and highlight its potential as a more effective digital marker.

In this study, for the first time, we found that TV can serve as a unique and novel digital marker for predicting MCI and dementia. Furthermore, gait parameters (GS, SL, TV) collected through intelligent wearable devices demonstrate exceptional predictive value for MCI and dementia. The significant correlation between TV and hippocampal atrophy as well as memory function confirms its potential as a novel gait marker for cognitive impairment. These findings lay the groundwork for future research on the early screening of MCI and dementia using smart wearable devices.

This study has several limitations. Firstly, it is a cross-sectional design, which means that we cannot establish the chronological order and determine causal inference. To confirm the observed results, follow-up visits with repeated measures are necessary. A longitudinal prospective design could be employed in future studies to investigate whether the decline in cognitive function precedes impaired turning function. Secondly, the measurement of the MTA level of the hippocampus should be improved in the future using automated analysis software to improve the accuracy of the experimental results. At last, the sample size in this study is relatively small, which may limit the generalizability of the findings. Insufficient power due to the small sample size can result in reduced reproducibility and potentially yield erratic or invalid results. Therefore, future studies should aim to include a larger study population to obtain more robust and reliable conclusions.

## 5 Conclusion

In our study, we have found that TV can be used as a distinctive and innovative digital indicator for predicting MCI and dementia. The strong correlation observed between TV and hippocampal atrophy as well as memory function, validates its potential as a novel gait marker for cognitive impairment. We are confident that our findings can contribute fresh insights for the development of future technology designs that aim to screen cognitive impairment in older adults.

## Data availability statement

The original contributions presented in the study are included in the article/Supplementary materials, further inquiries can be directed to the corresponding authors.

## Ethics statement

The studies involving humans were approved by the Ethics Committee of the First Affiliated Hospital of Soochow University. The studies were conducted in accordance with the local legislation and institutional requirements. The participants provided their written informed consent to participate in this study.

## Author contributions

JW: Data curation, Formal analysis, Investigation, Methodology, Project administration, Software, Supervision, Validation, Visualization, Writing – original draft. ZZ: Conceptualization, Data curation, Formal analysis, Investigation, Methodology, Project administration, Validation, Visualization, Writing – original draft. SC: Formal analysis, Investigation, Methodology, Project administration, Writing – review & editing. LZ: Conceptualization, Funding acquisition, Project administration, Resources, Supervision, Writing – review & editing. XS: Conceptualization, Project administration, Resources, Supervision, Writing – review & editing. ZS: Data curation, Investigation, Methodology, Project administration, Writing – review & editing. ZW: Data curation, Investigation, Methodology, Project administration, Writing – review & editing. JL: Conceptualization, Project administration, Resources, Supervision, Writing – review & editing. YQ: Conceptualization, Data curation, Methodology, Project administration, Resources, Supervision, Writing – review & editing. YW: Conceptualization, Data curation, Formal analysis, Funding acquisition, Investigation, Methodology, Project administration, Resources, Supervision, Validation, Visualization, Writing – review & editing.
