# Supplementary material for: Dual-task turn velocity – a novel digital biomarker for mild cognitive impairment and dementia
Source: Front Aging Neurosci. 2024 Feb 27;16:1304265. doi: 10.3389/fnagi.2024.1304265 (PMC10927999; doi:10.3389/fnagi.2024.1304265)
Supplement: Supplementary file 1 [file Data_Sheet_1.docx]

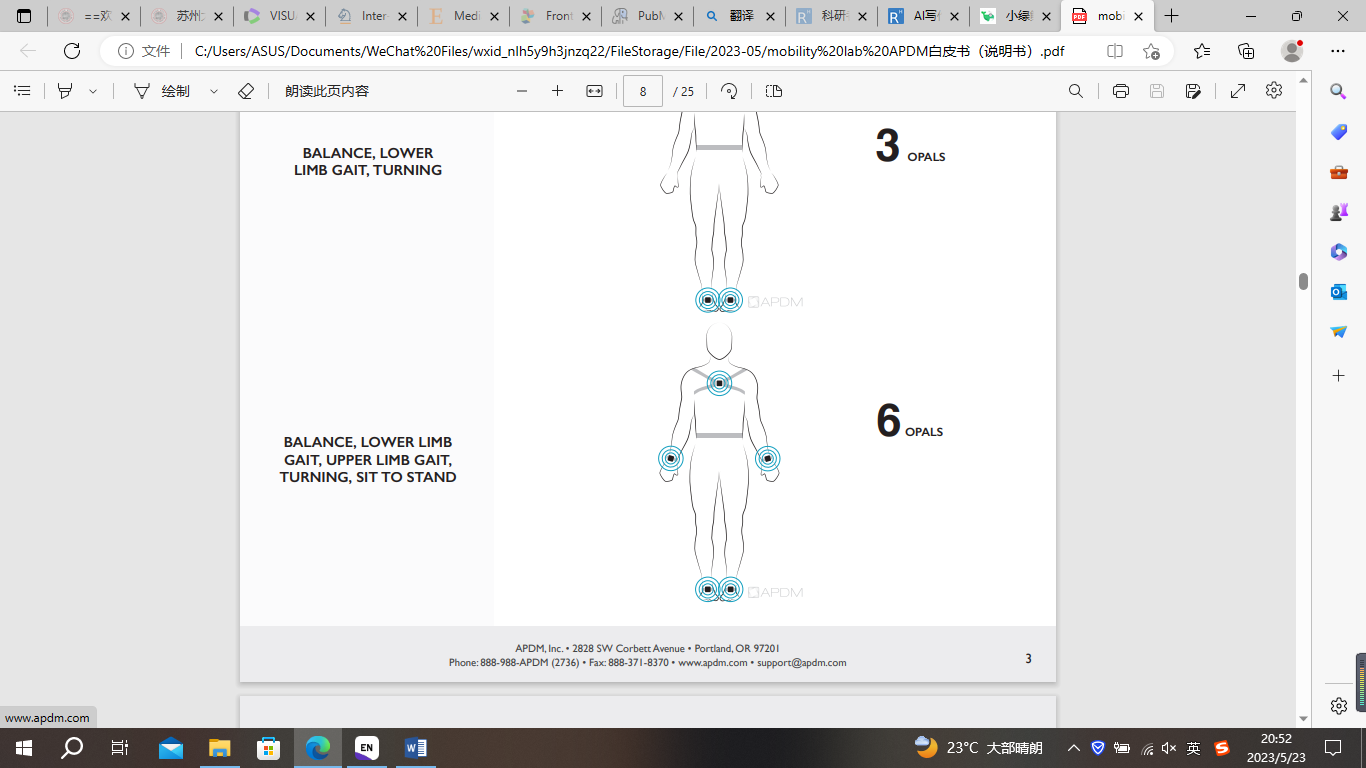

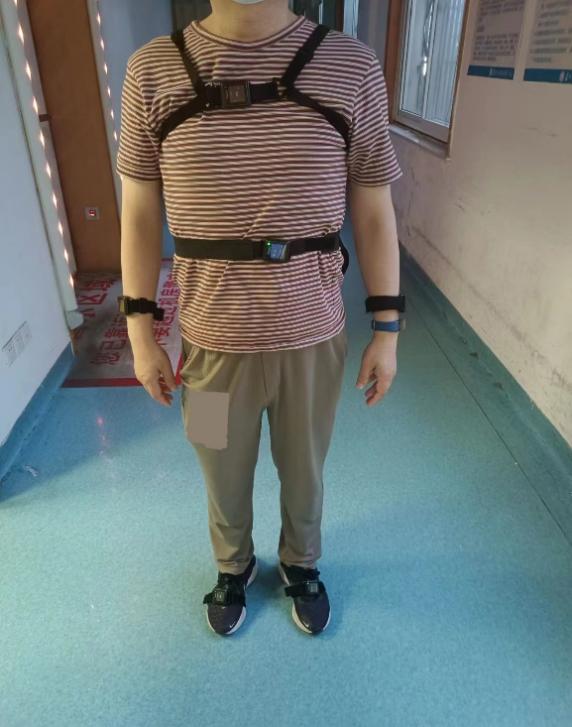


S1. The wireless APDM Movement Monitoring inertial sensor system, six opal inertial sensors (APDM Inc.) were worn on the wrists, ankles, sacrum, and chest during walking.

S2 Definitions of gait parameters.

| Gait Parameter Definitions | |
| --- | --- |
| Gait Speed | The forward distance (2 step lengths) travelled during the gait cycle divided by the gait cycle duration |
| Cadence | The number of steps per minute, counting steps made by both feet |
| Stride Length | The forward distance travelled by a foot during a gait cycle |
| Double Support | The percentage of the gait cycle in which both feet are on the ground |
| Lateral Step Variability | In three consecutive steps, the perpendicular deviation of the middle foot placement from the line connecting the first and the third step |
| Stance | The percentage of the gait cycle in which the foot is on the ground |
| Swing | The percentage of the gait cycle in which the foot is not on the ground |
| Turn Velocity | The peak angular velocity of the turn |


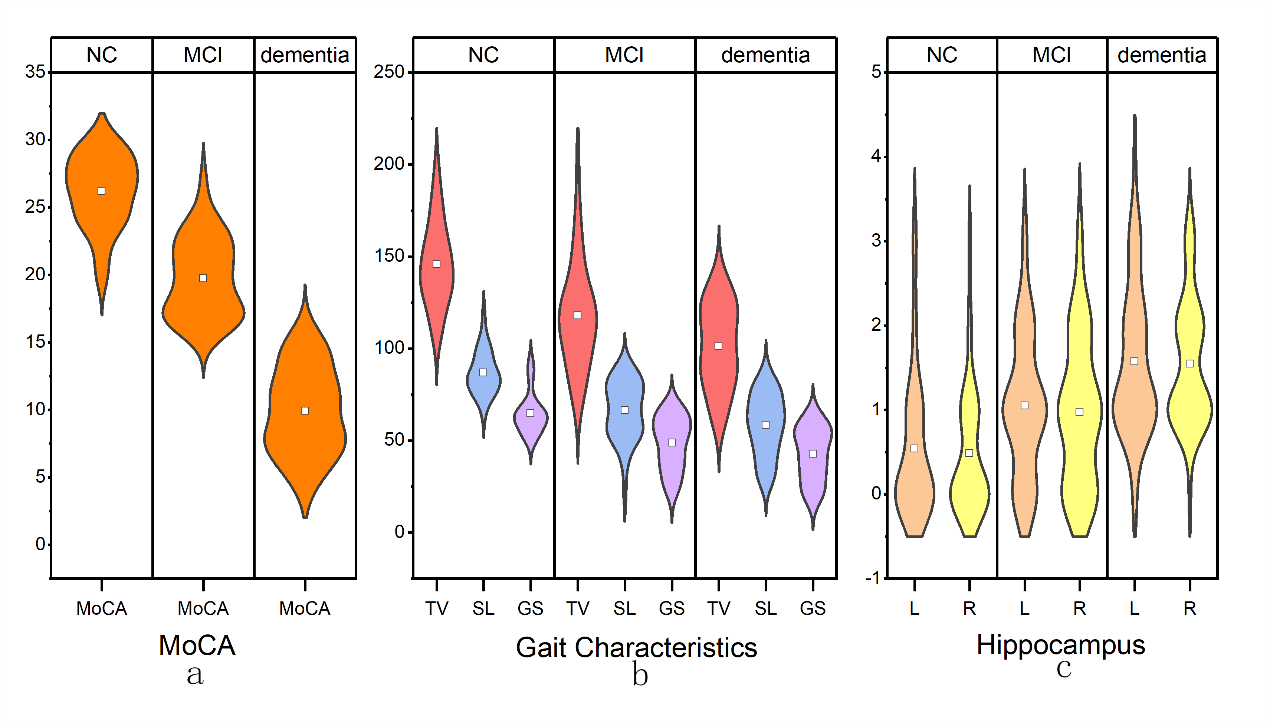


S3. (a).Distribution and mean of MOCA scores in three groups; (b).Distribution and mean of turn velocity(TV), stride length(SL), and gait speed(GS)under dual-task walking in three groups; (c).Distribution and mean of MTA scores of left and right hippocampus in three groups.

S4 Association of DT-gait characteristics with cognitive function in all participants

|  | Gait speed | | Stride length | | Turn velocity | |
| --- | --- | --- | --- | --- | --- | --- |
|  | r_s_ | *p* | r_s_ | *p* | r_s_ | *p* |
| MOCA | **0.429**** | 0.000 | **0.483**** | 0.000 | **0.478**** | 0.000 |
| Memory | **0.393**** | 0.000 | **0.415**** | 0.000 | **0.428**** | 0.000 |
| Attention | 0.260** | 0.004 | **0.347**** | 0.000 | 0.272** | 0.003 |
| Execution | -0.144 | 0.118 | -0.204* | 0.026 | **-0.278**** | 0.002 |
| Language | 0.309** | 0.001 | 0.325** | 0.000 | **0.392**** | 0.000 |

Adjusted for age、sex、years of education. MoCA:Montreal Cognitive Assessment;

S5 Multiple linear regression analysis of cognitive domains associated with DT-gait parameters of all participants

|  | Gait speed | | | | Stride length | | | | Turn velocity | | | |  |
| --- | --- | --- | --- | --- | --- | --- | --- | --- | --- | --- | --- | --- | --- |
|  | β | | *p* | | β | | *p* | | β | | *p* | |  |
| Memory | | 0.322 | | **0.003** | | 0.274 | | **0.007** | | 0.285 | | **0.006** | |
| Attention | | 0.048 | | 0.695 | | 0.166 | | 0.139 | | -0.014 | | 0.906 | |
| Execution | | 0.055 | | 0.538 | | -0.001 | | 0.994 | | -0.067 | | 0.434 | |
| Language | | 0.139 | | 0.212 | | 0.078 | | 0.443 | | 0.211 | | **0.048** | |

Adjusted for age、sex、years of education.

S6 Association of DT-gait characteristics with MTA scores of hippocampal volumes in all participants

|  | Gait speed | | Stride length | | Turn velocity | |
| --- | --- | --- | --- | --- | --- | --- |
|  | r_s_ | *p* | r_s_ | *p* | r_s_ | *p* |
| Hippocampal（L） | **-0.222*** | 0.014 | **-0.343**** | 0.000 | **-0.338**** | 0.000 |
| Hippocampal（R） | **-0.264**** | 0.003 | **-0.406**** | 0.000 | **-0.381**** | 0.000 |

S7 Characteristics of the study population(N=122)

| Variable | | NC（n=38） | MCI（n=42） | Dementia(n=42） |
| --- | --- | --- | --- | --- |
| AVLT-H |  | |  |  |
| Immediate recall | 16.2(4.1) | | **11.3(4.4) *** | **4.9(4.5) *^,^†** |
| Short delayed recall | 4.8(2.4) | | **2.6(2.5) *** | **0.9(2.1) *^,^†** |
| Long delayed recall | 4.4(2.7) | | **2.1(2.3) *** | **0.4(1.5) *^,^†** |
| DST |  | |  |  |
| Forward | 7.9(0.8) | | **7.1(1.0) *** | **5.8(1.2) *^,^†** |
| Backward | 4.0(1.1) | | **3.3(0.9) *** | **2.0(1.4) *^,^†** |
| SCWT |  | |  |  |
| Dot(s) | 24.5(9.1) | | **31.3(10.6) *** | **38.0(14.0) *^,^†** |
| Words(s) | 29.4(10.7) | | **40.4(21.5) *** | **47.9(22.2) *** |
| Color(s) | 39.7(1.9) | | **56.2(30.8) *** | **62.9(24.7) *** |
| Gait characteristics (Single-task) | | |  |  |
| Cadence (steps/min) | 101.8(9.5) | | 103.1(10.9) | 101.2(12.7) |
| Double support L(%GCT) | 23.4(3.1) | | 25.3(4.4) | **26.5(5.5) *** |
| Double support R(%GCT) | 23.5(3.0) | | 25.1(4.3) | **26.6(5.5) *** |
| Lateral step variability(cm) | 7.9(2.8) | | 6.7(3.1) | **6.0(2.6) *** |
| Stance(%GCT) | 61.6(1.6) | | 62.5(2.2) | **63.3(2.7) *** |
| Swing(%GCT) | 38.3(1.6) | | 37.5(2.2) | **36.7(2.7) *** |

NC: normal cognitive; MCI: mild cognitive impairment; AVLT-H: auditory verbal learning test-HuaShan version; DST: digital span test; SCWT: Stroop color word test ;******P*＜0.05,versus NC; **†***P*＜0.05,versus MCI;
